# Thoracoscopic resection of pulmonary sequestration with carbon dioxide insufflation and indocyanine green

**DOI:** 10.1093/icvts/ivac209

**Published:** 2022-08-10

**Authors:** Sachie Koike, Masahisa Miyazawa, Nobutaka Kobayashi

**Affiliations:** Department of Thoracic Surgery, Japan Red Cross Society Nagano Hospital, Nagano, Japan; Division of General Thoracic Surgery, Department of Surgery, Shinshu University School of Medicine, Matsumoto, Japan; Department of Thoracic Surgery, Japan Red Cross Society Nagano Hospital, Nagano, Japan; Department of Thoracic Surgery, Japan Red Cross Society Nagano Hospital, Nagano, Japan

**Keywords:** Pulmonary sequestration, Supplied from renal artery, Carbon dioxide insufflation, Indocyanine green

## Abstract

We encountered a rare case of pulmonary sequestration supplied from the right renal artery, which was resected by video-assisted thoracic surgery with carbon dioxide insufflation and indocyanine green-guided technique. A 41-year-old woman with intralobar pulmonary sequestration supplied from the right renal artery was referred to our department. At the time of surgery, we used carbon dioxide insufflation to improve the manoeuvrable workspace for shutting off aberrant arteries and indocyanine green fluorescence guidance to differentiate the boundary of the sequestered lung from the normal lung. These procedures helped in the efficient resection of the lesion.

## INTRODUCTION

Pulmonary sequestration is a rare congenital malformation characterized by a mass of non-functioning pulmonary tissue that lacks normal communication with the tracheobronchial tree and receives aberrant blood supply from the systemic arteries [1]. The gold standard of treatment is resection of the sequestrated lung and shutting off aberrant arteries [2]. Here, we report a very rare case of intralobar pulmonary sequestration supplied from the right renal artery, which was successfully resected by video-assisted thoracic surgery (VATS) using carbon dioxide (CO_2_) insufflation and indocyanine green (ICG).

## CASE REPORT

**Figure 1:**
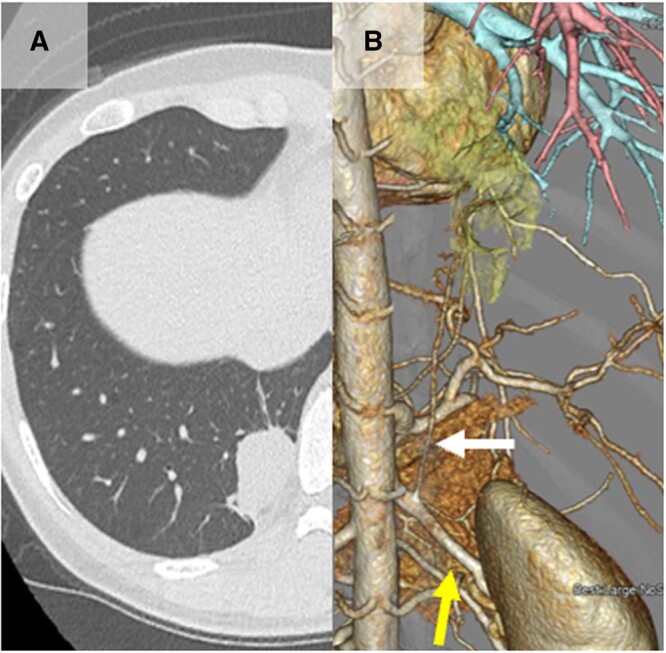
(**A**) Sequestered lung in dorsobasal segment (S10) of the right lower lobe as a isodensity mass on computed tomography. (**B**) Three-dimensional computed tomography showing an aberrant artery (white arrow) arising from the right renal artery (yellow arrow) (A color version of this figure appears in the online version of this article).

A 41-year-old woman was referred to us after a mass in the right lower lobe of the lung was detected on computed tomography. A 5.5-cm mass was visualized in the dorsobasal segment (S10) of the right lower lobe of the lung (Fig. 1A). On three-dimensional computed tomography, an aberrant artery originating from the right renal artery (Fig. 1B), and the lesion lacked normal communication with the tracheobronchial tree was observed. Therefore, she was diagnosed as intralobar pulmonary sequestration and scheduled to undergo VATS.

**Figure 2:**
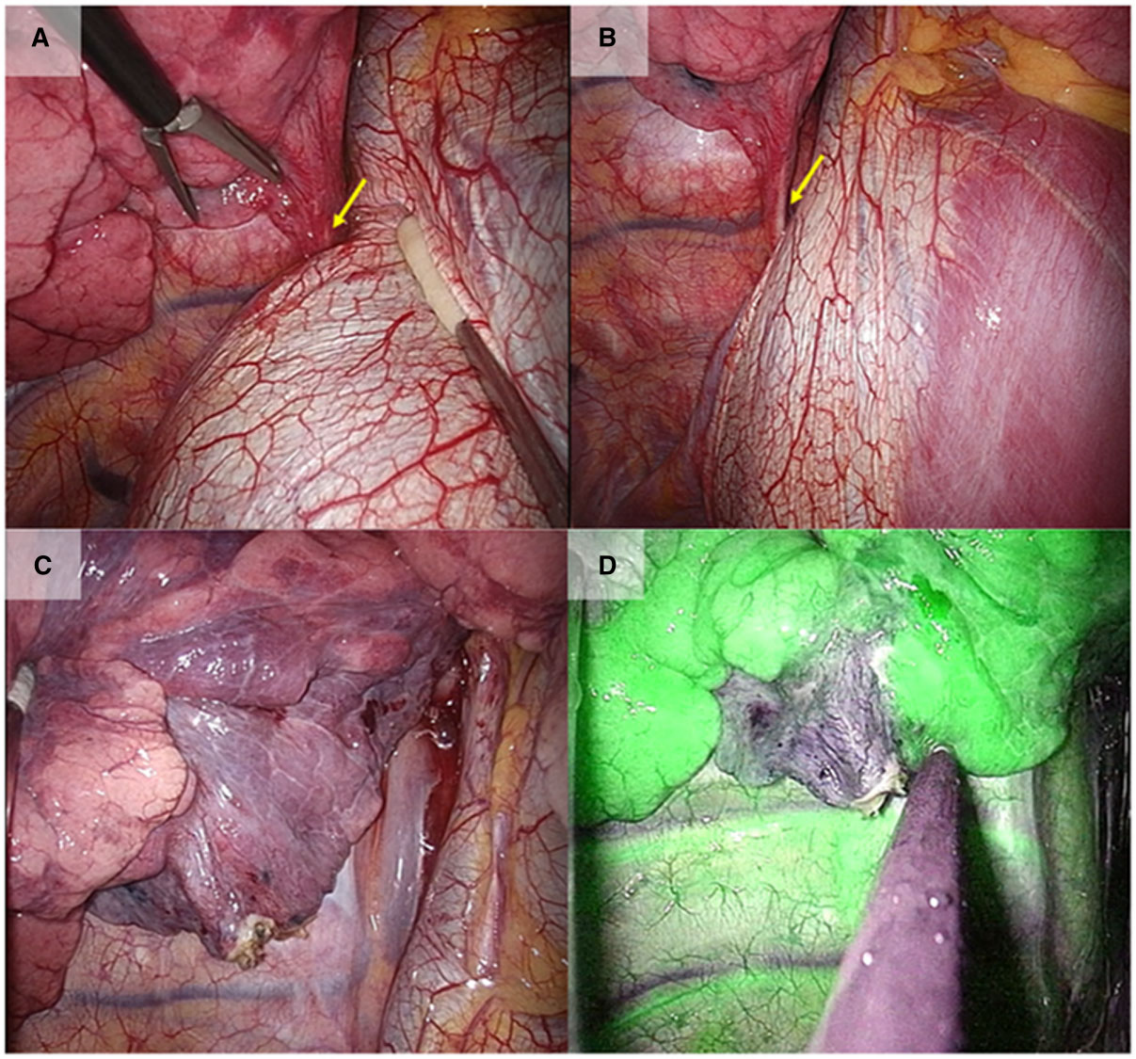
(**A**) An aberrant artery comes from the right renal artery just above the diaphragm (yellow arrow). (**B**) After carbon dioxide insufflation, the diaphragm was almost flattened, which increased the workspace dramatically. (**C**) The boundary between normal lung and sequestrated lung was unclear in the normal thoracoscopic view. (**D**) Fluorescence navigation using indocyanine green showing green staining in the normal lung and no staining in the sequestrated lung. The boundary clarified. (A color version of this figure appears in the online version of this article).

During the operation, we found an aberrant artery ascending through the right hemidiaphragm and coursing through the leaves of the lowermost edge of the pulmonary ligament which was managed to obtain surgical field with compression of the diaphragm (Fig. 2A). By CO_2_ insufflation (8 mmHg), the diaphragm became nearly flat and the workspace improved without requiring its compression (Fig. 2B). We divided the elastic aberrant artery using LigaSure vessel sealing systems (Medtronic, MN, USA) following hemoclip ligation (Teleflex Medical, North Carolina, USA) to prevent stump Haemorrhage. The boundary between the normal lung and the lesion was unclear by the normal thoracoscopic view (Fig. 2C). An anaesthesiologist intravenously administered 0.25 mg/kg ICG, and then, we used real-time fluorescent imaging technique with VISERA ELITE II (Olympus, Japan) to clarify the boundary. The demarcating margin between the sequestrated and normal lungs was identified and marked using electrocautery (Fig. 2D). The sequestrated lung was stapled based on the boundary line. Her postoperative course was uneventful.

## COMMENT

The mainstay of treatment of pulmonary sequestration surgical resection, and the procedures are resection of the sequestrated lung and shutting off aberrant feeding artery. The difficulty of resecting a pulmonary sequestration is identifying the aberrant artery [2]. In our case, because the aberrant artery originated from the right renal artery, it was in the lowermost edge of the pulmonary ligament, just above the diaphragm. We were able to get enough workspace by flattening the diaphragm with CO_2_ insufflation and to shut off aberrant feeding artery safely. CO_2_ insufflation has been used in VATS to improve the work space for surgical manoeuvrability near diaphragm [3]. Our experience showed the utility of CO_2_ insufflation for shutting off the aberrant artery of pulmonary sequestration.

Identifying the precise margins of the sequestrated lung is another necessary step for resection of the sequestrated lung. Initially, we could not differentiate the boundary between the sequestrated lung and the normal lung in the normal thoracoscopic view. Hence, we used intraoperative ICG guidance to clarify the boundary and perfom resection without any loss of normal lung parenchyma, Although, this may be difficult in cases inflammation or adhesions [4]. Several cases of resection of intralobar pulmonary sequestration using ICG have been reported [4, 5].

We report a rare case of intralobar pulmonary sequestration in which an aberrant artery was supplied from right renal artery. CO_2_ insufflation and intravenous ICG administration were useful.

**Conflict of interest:** none declared.

## Reviewer information

Interactive CardioVascular and Thoracic Surgery thanks Tahir Sevval Eren, Jozsef Furak and the other, anonymous reviewer(s) for their contribution to the peer review process of this article.
